# Identification of Critical Amino Acids Conferring Lethality in VopK, a Type III Effector Protein of *Vibrio cholerae*: Lessons from Yeast Model System

**DOI:** 10.1371/journal.pone.0141038

**Published:** 2015-10-21

**Authors:** Leela Krishna Bankapalli, Rahul Chandra Mishra, Balvinder Singh, Saumya Raychaudhuri

**Affiliations:** Institute of Microbial Technology, Council of Scientific and Industrial Research, Molecular Biology Division, Chandigarh, India; Centre National de la Recherche Scientifique, Aix-Marseille Université, FRANCE

## Abstract

VopK, a type III effector protein, has been implicated in the pathogenesis of *Vibrio cholerae* strains belonging to diverse serogroups. Ectopic expression of this protein exhibits strong toxicity in yeast model system. In order to map critical residues in VopK, we scanned the primary sequence guided by available data on various toxins and effector proteins. Our in silico analysis of VopK indicated the presence of predicted MCF1-SHE (SHxxxE) serine peptidase domain at the C-terminus region of the protein. Substitution of each of the predicted catalytic triad residues namely Ser^314^, His^353^ and Glu^357^ with alanine resulted in recombinant VopK proteins varying in lethality as evaluated in yeast model system. We observed that replacement of glutamate^357^ to alanine causes complete loss in toxicity while substitutions of serine^314^ and histidine^353^ with alanine exhibited partial loss in toxicity without affecting the stability of variants. In addition, replacement of another conserved serine residue at position 354 (S^354^) within predicted S^314^H^353^E^357^ did not affect toxicity of VopK. In essence, combined in silico and site directed mutagenesis, we have identified critical amino acids contributing to the lethal activity of VopK in yeast model system.

## Introduction

*Vibiro cholerae*, causative agent of the life threatening diarrheal disease, cholera, possesses a multitude of virulence associated factors whose collective action aids the organism in becoming a successful pathogen. Of late, the type III secretion system (T3SS) and several effector proteins which are delivered by this specialized transport machinery have been implicated in the pathogenesis of *Vibrio cholerae* strains belonging to diverse serogroups [1]. In recent years, much progress has been made in characterizing various T3SS effector proteins of *Vibrio cholerae*. Of these, VopF has received much of the attention. By employing yeast, tissue culture and animal model systems, many crucial facts on the structural and functional aspects of VopF have been garnered [2–7]. In addition to VopF, the existence of additional 11 effector proteins has been revealed in the T3SS island of *Vibrio cholerae* strain AM-19226 [5]. Of these, VopX and VopK exhibit strong lethality in yeast model system [5]. Using yeast model system, VopX has been shown to interact with components of the cell wall integrity mitogen-activated protein kinase (MAPK) pathway. Interestingly, VopK (locus number A33_1699) shares homology with Mcf (makes caterpillars floppy), an insecticidal toxin of *Photorhabdus luminescens* and it is also known as McfV [8].

In recent years, T3SS and effector proteins (T3Es) delivered by such export machinery directly into host cytosol have consumed much attention. As a consequence, a great deal of work has been attributed to gain structural and functional insights of numerous effector proteins. There is now compelling evidence how evolution has incorporated eukaryotic motif and domains to many effector proteins and toxins [9, 10], thereby enabling pathogens to usurp host cellular machinery by employing such specialized virulence factors.

Until now, limited information is available in the literature about the structural and functional aspects of VopK. In this work, we wished to investigate the presence of a functional unit and evaluate its contribution to the toxic activity of VopK. VopK shows some homology (29% identity, 45% positives) with the 136-amino acid region preceding the BH3 domain in Mcf, an insecticidal toxin [8]. Recently, Aravind and co-workers have identified a novel serine peptidase catalytic centre otherwise known as MCF1-SHE domain in Mcf toxin [9]. As illustrated, the MCF1-SHE catalytic core comprises two kinked helices, which are associated with a conserved serine and a HxxxE motif [9]. Incidentally, this domain is frequently observed at the extreme C-terminus of many polymorphic toxins (e.g entomotoxins from *Pseudomonas fluorescens*) as well as secreted proteins from various intracellular and extracelluar pathogens, further potentiating its role in the toxic activity of such toxins [9, 11]. Armed with the available information, we scanned the primary sequence of VopK. Our in-silico analyses predicted a MCF1-SHE domain and catalytic triad residues (Ser^314^/His^353^/Glu^357^) in VopK. In addition, we also identified another conserved serine residue at position 354 within the predicted SHE (Ser^314^/His^353^/Glu^357^) domain. Combined site-directed mutagenesis and yeast model system helped us to evaluate the contribution of these residues within the predicted MCF1-SHE domain of VopK where substitution of glutamate with alanine at position 357 (Glu^357^Ala) completely jeopardized the function whereas substitutions of serine and histidine at positions 314 and 353 respectively resulted a partial loss of toxicity of this effector protein. Replacement of serine at position 354 with alanine did not affect the functionality of VopK.

## Materials and Methods

### Bacterial strains and media

The strains and plasmids used in this study are listed in Table 1. *Escherichia coli* Nova blue (Novagen) was used for general cloning purpose. The *E*. *coli* strain was propagated at 37°C in liquid with agitation or on solid (1.5% agar) in Luria Broth unless mentioned otherwise. *Escherichia coli* BL21 (DE3) (Novagen) was used for the over expression of protein. *Saccharomyces cerevisiae* strain BY4741 (*MATa; his3Δ 1; leu2Δ 0; met15Δ 0; ura3Δ 0*) used in this study (Table 1) was grown in YPD (1% (w/v) yeast extract, 2% (w/v) peptone, 2% (w/v) glucose) broth or agar (2%).

**Table 1:** Strains and Plasmids used in this study.

Strains/Plasmids	Genotype/Description	Source
***E*. *coli* Strains**		
BL21 (DE3)	*E*.*coli B*, *F* ^-^ *ampT lon*, with a λ prophage carrying the T7 RNA polymerase	Novagen
NovaBlue	*E*. *coli* K-12, *recA endA*, *lacI^q^*,*lacY*	Novagen
***Saccharomyces cerevisiae* strains**		
BY4741	*MATa; his3Δ 1; leu2Δ 0; met15Δ 0; ura3Δ 0*	This study
BY4741—LEU	BY4741 + pESC-LEU vector, leu2 selection	This study
BY4741—LEU-VopK	BY4741 + pESC-LEU-VopK, LEU2 selection	This study
BY4741—LEU-VopK^S314A^	BY4741 + pESC-LEU-VopK^S314A^, LEU2 selection	This study
BY4741—LEU-VopK ^H353A^	BY4741 + pESC-LEU-VopK^H353A^, LEU2 selection	This study
BY4741—LEU-VopK ^E357A^	BY4741 + pESC-LEU-VopK^E357A^, LEU2 selection	This study
BY4741—H10	BY4741 + pGMH10 vector, HIS3 selection	This study
BY4741—H10—VopK	BY4741 + pGMH10—VopK, HIS3 selection	This study
BY4741—H10—VopK^S314A^	BY4741 + pGMH10—VopK^S314A^, HIS3 selection	This study
BY4741—H10—VopK^H353A^	BY4741 + pGMH10—VopK^H353A^, HIS3 selection	This study
BY4741—H10—VopK^E357A^	BY4741 + pGMH10—VopK^E357A^, HIS3 selection	This study
BY4741—H10—VopK^S354A^	BY4741 + pGMH10—VopK^S354A^, HIS3 selection	This study
**Plasmids**		
pESC-LEU	LEU2 selection	Agilent Technologies
pGMH10	HIS3 selection	RIKEN
pET15b	Ap^r^, N-terminal 6His-tag expression vector	Novagen
pESC-LEU-VopK	1.275 kb *VopK* gene fragment amplified from genomic DNA with XhoI—NheI VopK primers by PCR and cloned into pESC—LEU which was digested with same restriction enzymes	This study
pGMH10-VopK	1.275 kb *VopK* gene fragment amplified from genomic DNA with BamHI-SmaI VopK primers by PCR and cloned into pGMH10 which was digested with same restriction enzymes	This study
pESC-LEU-VopK^S314A^	VopK harboring A in place of S at position 314	This study
pESCL-EU-VopK ^H353A^	VopK harboring A in place of H at position 353	This study
pESC-LEU-VopK ^E357A^	VopK harboring A in place of E at position 357	This study
pGMH10-VopK^S314A^	VopK harboring A in place of S at position 314	This study
pGMH10-VopK^H353A^	VopK harboring A in place of H at position 353	This study
pGMH10-VopK^E357A^	VopK harboring A in place of E at position 357	This study
pGMH10-VopK^S354A^	VopK harboring A in place of S at position 354	This study

### Construction of strains and alanine substitution mutagenesis of putative SHE catalytic triad of VopK

The *vopK* gene was amplified from the genomic DNA of *Vibrio cholerae* strain SC110 using the primers XhoI VopK and NheI VopK. After verifying the fragment by sequencing, it was cloned into the XhoI /NheI sites of pESC-LEU (Agilent Technologies) under the control of the GAL1 promoter. According to manufacturer’s guidelines, ORF cloned in this vector will be expressed as a tagged protein with an N-terminal Myc-tag. Positive clones were further confirmed by sequencing and maintained in *E*. *coli* strain NovaBlue cells. The selected construct, designated as pESC-LEU-VopK, was transformed into *S*. *cerevisiae* strain BY4741. The resultant strain was marked as BY4741- LEU-VopK.

The recombinant plasmid pESC-LEU-VopK was used as a template for alanine mutagenesis reactions. The alanine substitution of S^314^H^353^E^357^ residues was performed using Stratagene one step mutagenesis kit. All the constructs were sequenced in their entirety to confirm the clones and the desired mutations at the corresponding positions. The defined plasmids were further transformed into BY4741 to generate a series of recombinant *S*. *cerevisiae* strains (Table 1). In addition, we have also cloned wild type VopK and alanine variants of S^314^H^353^E^357^S^354^ in BamHI /SmaI sites of pGMH10, low copy number vector (RIKEN) under the control of the GAL1 promoter (Table 1). According to the manufacturer’s guidelines, ORF cloned in this vector will also be expressed as a tagged protein with a C-terminal Myc-tag. Positive clones were further confirmed by sequencing and maintained in *E*. *coli* strain NovaBlue cells. The desired construct, designated as pGMH10-VopK, was retransformed into *S*. *cerevisiae* strain BY4741. The resultant strain was marked as BY4741-H10-VopK.

### Purification and proteolytic assay of VopK

VopK protein was purified by Ni^2+^-nitrilotriacetic acid (Ni^2+^-NTA) chromatography by following a published protocol [12]. The wild-type gene was cloned into the NdeI-BamHI site of the pET15b vector (Novagen) to generate an N-terminal His_6_-VopK fusion protein. The clone was confirmed by sequencing and transformed into *E*. *coli* BL21 (DE3). After induction with 0.4mM IPTG (isopropyl1-thio-β-D-galactopyranoside), VopK protein was purified through Qiagen Ni^2+^-NTA column. The purified protein was dialyzed overnight in a solution of buffer A containing 10mM Tris, pH 7.9, 100 mM KCl, 0.1mM EDTA, 0.1mM DTT, 5% glycerol. Protease assay was done essentially as described earlier [13].

### Yeast growth assay

The wild type and the corresponding alanine derivatives of S^314^H^353^E^357^S^354^ residues of VopK were transformed into *S*. *cerevisiae* strain BY4741. The transformed yeast strains (Table 1) were grown in synthetic selective medium (SC) containing 0.17% (w/v) yeast nitrogen base without amino acids, 0.5% (w/v) ammonium sulphate, 2% (w/v) glucose and was supplemented with appropriate amino acids and nucleic acid bases. SC^Gal^ and SC^Raf^ were SC media containing 2% (w/v) galactose or raffinose, respectively instead of glucose. All the recombinant yeast strains were subjected to galactose induction experiment essentially as described previously [4] with modification [4, 14]. Briefly, overnight cultures of all recombinant strains in SC^Raf^ medium were diluted and grown again in SC^Raf^ medium at 30°C to exponential phase till OD_600_ was 0.8–1.0. Effect of the expression of VopK and alanine derivatives on the growth of *S*. *cerevisiae* BY4741 was examined by spotting equal number of cells onto SC and SC^Gal^ plates lacking the corresponding auxotrophic markers to maintain the plasmids. Growth was monitored after 60-70h at 30°C and photographed accordingly. Liquid growth assay was done as described earlier [7, 15]. Briefly, overnight cultures of recombinant yeast strains harboring wild type and alanine variants of VopK grown in raffinose were diluted back to an OD_600_ of 0.05 in 25 ml of indicated media with appropriate amino acids supplementation. The absorbance was measured at 600nm for stipulated period.

### Preparation of Yeast Extracts and Immunoblot Analysis

The immunoblot analysis was essentially done by adopting a published protocol [7]. All recombinant yeast cells were grown in SC^Raf^ medium at 30°C till mid-log phase. The cultures were then diluted directly in induction media (SC^Gal^). After 6 hours of induction, cultures were pelleted in a refrigerated centrifuge. Cells were lysed in 150μl of breaking buffer (50mM Tris-HCl (pH 7.5), 10% glycerol, 1% Triton X-100, 0.1% SDS, 150mM NaCl, 50mM NaF, 1mM Sodium Orthovandate, 50mM β-Glycerol phosphate, 5mM EDTA, 1mM phenylmethylsulfonylfluoride and the 1X protease inhibitor cocktail by vigorous vortex with 0.3-mm glass beads (Sigma)). Cell extracts were separated from glass beads and cell debris, collected in a new centrifuge tube by centrifugation, and further clarified by a 13,000 x *g* spin for 15 min at 4°C. The protein concentration of the supernatants was measured by BCA Protein assay kit (Pierce). Equal concentration of protein samples were fractionated by SDS-polyacrylamide gel electrophoresis using 12% polyacrylamide gels and transferred to Immobilon—P Transfer Membrane (Millipore). Membranes were probed with either anti-myc monoclonal antibody (Thermo Scientific) or anti-actin monoclonal antibody (Millipore). The primary antibody was detected using a horseradish peroxidase conjugated anti-rabbit antibody with the Millipore detection system.

## Results and Discussion

### Expression of VopK is lethal to yeast model system

**Fig 1:**
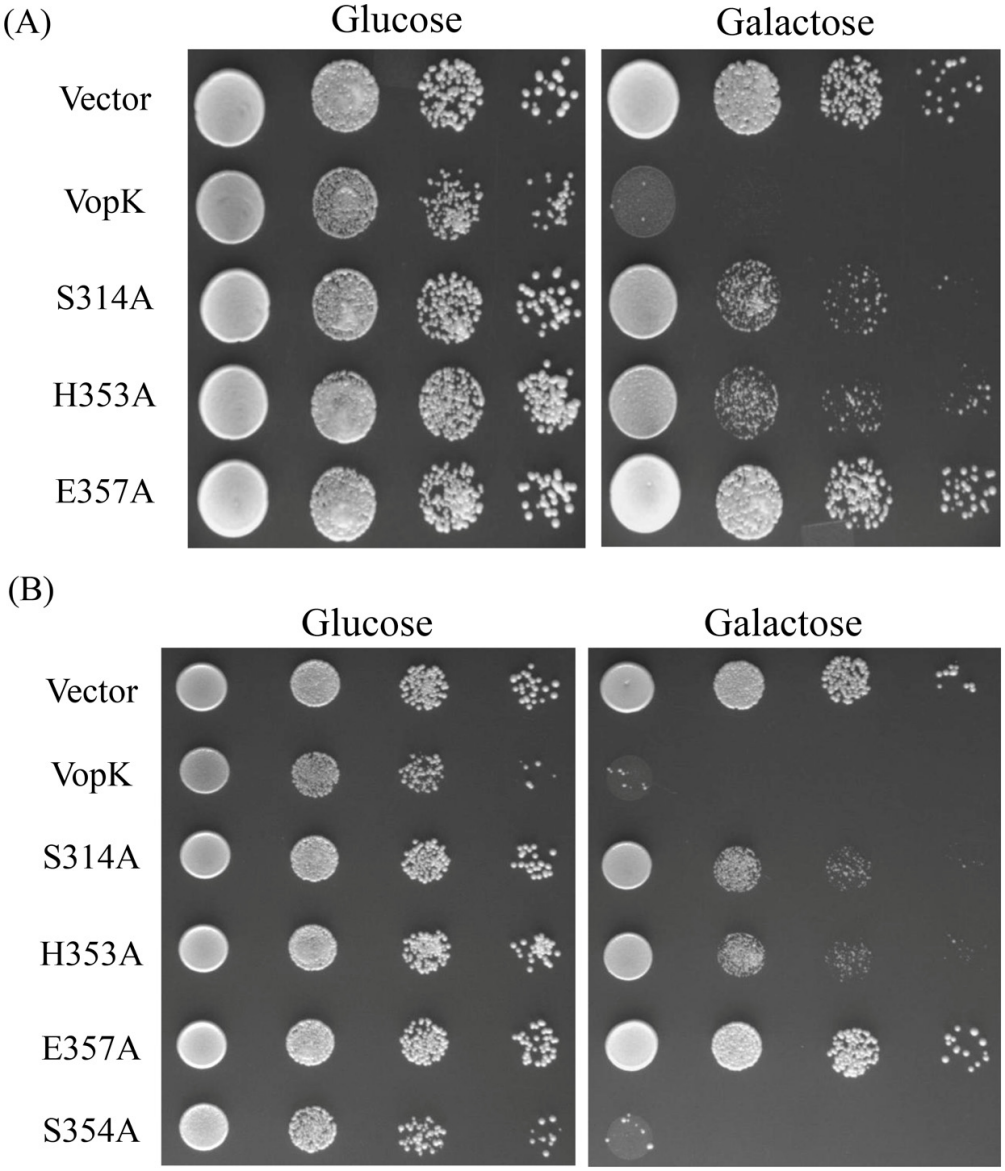
Yeast growth inhibition assay. The growth of yeast strain BY4741 expressing wild type and mutant proteins of VopK under the control of GAL1 promoter from pESC-LEU (A) or pGMH10 (B). Cells transformed with empty vector alone were used as a negative control. The indicated strains were grown overnight in non-inducing selective synthetic medium (SC^raf^) containing 2% raffinose as the carbon source. Equal number of cells were spotted at 10° and three serial dilutions of 10^−2^, 10^−3^ and 10^−4^ (left to right) on SC^Glu^ (left panel, repression) and SC^Gal^ (induction) agar. The data shown are representative of three independent experiments.

There is now a growing appreciation on the utility of *Saccharomyces cerevisiae*, the budding yeast as a non-mammalian model system to identify and evaluate functionality of diverse arrays of virulence factors [14, 16–21]. Previously, our group has also exploited the utility of this yeast model system for gathering functional insights of another *Vibrio cholerae* effector protein [4, 7]. To examine the lethality of VopK, we expressed VopK ectopically under the control of GAL promoter in *S*. *cerevisiae* strain BY4741 from pESC-LEU, a high copy number vector (Table 1). Yeast cells transformed with pESC-LEU-VopK (Table 1) yielded a strong lethal phenotype under inducing condition (Fig 1A). Our spotting data is also in congruence with previous observation made by Dziejman and colleagues [5]. We also observed weak growth inhibition under repressing (glucose) condition (Fig 1A). This can be explained in terms of leaky expression from GAL1 promoter of pESC-LEU vector. The leaky expression problem associated with GAL1/10 promoter from different vectors has also been documented in earlier studies [20–22].

Earlier studies have clearly demonstrated that low level expression of effector proteins increases the specificity while high level expression promotes sensitivity of inhibition in yeast model system [20]. As evidenced, several effector proteins exhibit toxicity only when expressed at high level [20]. In some cases, it is also surmised that high level expression may result in non-specific toxic effects [23]. We wanted to ascertain whether low level expression of VopK maintains similar toxicity as presented in the preceding section, the gene encoding wild type VopK was cloned in pGMH10, a low copy number vector (Table 1). Yeast cells transformed with pGMH10-VopK (Table 1) yielded a lethal phenotype only in inducing condition (Fig 1B).

### Identification of a predicted MCF1-SHE domain in VopK

**Fig 2:**
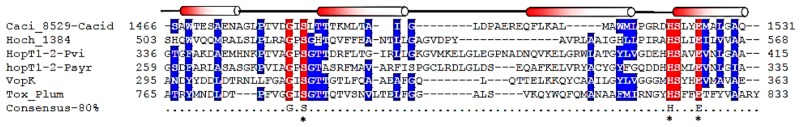
Multiple sequence alignment of MCF1-SHE domain proteins. Alignment of VopK along with other polymorphic toxins and effector proteins as carried out using T-coffee [36]. The conserved and predicted SHE motif is marked with star (_*_). Identical and similar regions are shown highlighted as red and blue, respectively. Helices in the secondary structure of VopK as predicted by PSI-PRED [37, 38] are shown as cylinders (red and white gradient) along with coils as lines (black). Secondary elements of VopK are overlapping with those of other proteins as shown by Arvind and colleagues [9]. Accession numbers are as follows: ACU77352,Caci_8529 [*Catenulispora acidiphila* DSM 44928]; ACY13938, Hoch_1384 [*Haliangium ochraceum* DSM 14365]; EEA94476, HopT1-2 [*Pseudovibrio* sp. JE062]; EEB62143, hopT1-2 [*Pseudomonas syringae pv*. *tomato* T1]; EDN16474, VopK A33_1699 [*Vibrio cholerae* AM-19226]; AAM88787, Toxin protein [*Photorhabdus luminescens*].

The recent flurry of articles has produced a rich harvest of data that exudes how diverse bacterial effector proteins have evolved with eukaryotic domains and motifs, thus enabling pathogens to hijack and evade host surveillance system [10]. To evaluate whether VopK (McfV) contains any functional unit contributing to its lethality, we carried out sequence analysis with PSI-BLAST (default parameters) using VopK as query. Subsequently, a multiple alignment of VopK with polymorphic toxins and T3SS effector proteins reveals the presence of an MCF1-SHE domain in VopK (Fig 2). The predicted MCF1-SHE domain in VopK has conserved Ser^314^, His^353^ and Glu^357^ residues where His^353^ and Glu^357^ form the characteristic HxxxE motif (Fig 2). Further analysis of secondary structure also confirms that the positional distribution of these residues is similar to that in known MCF1-SHE domain containing toxins and effector proteins (Fig 2, [9]). We also noticed a glycine residue (Gly ^315^) in close proximity to the serine residue (Ser ^314^) (Fig 2), a criterion to satisfy the presence of a serine protease catalytic triad [24].

**Fig 3:**
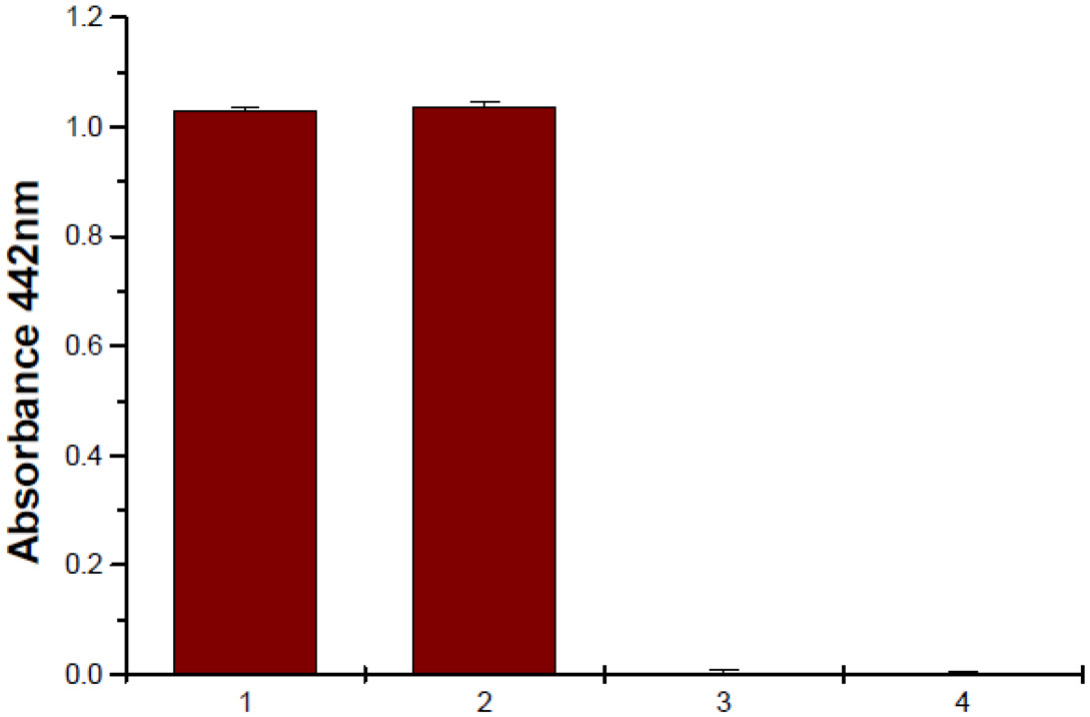
Protease assay. Purified VopK protein of different amounts was subjected to proteolytic activity using azocasein as a substrate. Proteinase K treated as a control (1, 2). 1: proteinase K 50μg; 2: proteinase K 100μg; 3: VopK 50μg; 4: VopK 100μg

As demonstrated in the preceding section that VopK harbors catalytic triads further prompted us to evaluate protease activity of the protein under in-vitro condition. In this regard, recombinant VopK was purified by Ni^+2^ –nitriloacetic acid (Ni-NTA) chromatography and subjected to protease assay using azocasein as substrate as described elsewhere [13]. Contrary to our expectation, no protease activity was detected with VopK under this condition (Fig 3). Similar observation was also noticed by Nimchuck and colleagues where HopX family effector proteins despite of having strong homology and conservation of PNGase-like catalytic triad residues failed to exhibit any detectable enzymatic activity on commonly tested substrates under in vitro condition [25]. It is also evidenced from other studies that numerous type III effecotrs belonging to family of cysteine proteases and protein kinases become enzymatically active under in vitro condition only in the presence of an in vivo host target or such enzymes require processing to become active or both [11, 26–29]. Thus far, we have surmised that an MCF1-SHE serine peptidase domain has been predicted and the related catalytic triad residues (Ser^314^/His^353^/Glu^357^) have been identified in VopK. Based on our in vitro protease assay, we concluded that protease activity of VopK may require the presence of in vivo substrates and or host activators.

### Role of predicted Ser^314^/His^353^/Glu^357^ catalytic triad residues in the lethality of VopK by employing yeast as a model system

To determine the significance of S^314^H^353^E^357^ residues in the functioning of VopK, the selected amino acids were substituted with alanine residues and the corresponding alanine variants were transformed into *S*. *cerevisiae* strain BY4741. The recombinant yeast strains were subjected to solid agar spotting assay on SC and SC^Gal^. The growth of each recombinant yeast strain in the dilution spotting assay is used as a marker to evaluate the extent of functional alteration of each VopK- SHE alanine variant. This was done according to the published protocol [7]. For example, any recombinant yeast strain with a VopK variant protein that exhibits growth at the highest dilution as well as scores equal to or greater than 70% of growth restoration as compared to vector control (calculated on the basis of mean area density as compared to vector control) is considered as demonstrating full restoration of growth. Conversely, this indicates complete loss of lethality in the corresponding VopK variant protein harbored by that recombinant yeast strain. If percentage of growth restoration falls within the range of 30–70%, it is considered to display partial restoration of growth, which further suggests partial loss in toxicity of corresponding VopK variant protein. Our data suggested that the substitution of glutamate^357^ with alanine resulted in complete loss of toxicity of VopK while replacements of histidine^353^ and serine^314^ with alanine showed partial loss of lethality as evaluated by growth of recombinant yeast strains according to the parameters described earlier (Fig 1A, [7]).

To investigate whether alanine variants of SHE triads (Ser^314^Ala/His^353^Ala/Glu^357^Ala) of VopK exert similar toxicity as presented in the preceding section (Fig 1A) under low level expression, we reconstructed recombinant clones of SHE alanine variants in pGMH10, a low copy number vector (Table 1). All recombinants clones in this vector were subsequently transformed into *S*. *cerevisiae* strain BY4741 and subjected to solid agar spotting assay. We observed similar pattern of growth where VopK exhibited its strong lethality while substitution of glutamate at position 357 with alanine completely abrogated toxicity of VopK-E357A variant and both VopK-S314 and VopK-H353 maintained partial toxicity as enumerated by published method (Fig 1B, [7]).

**Fig 4:**
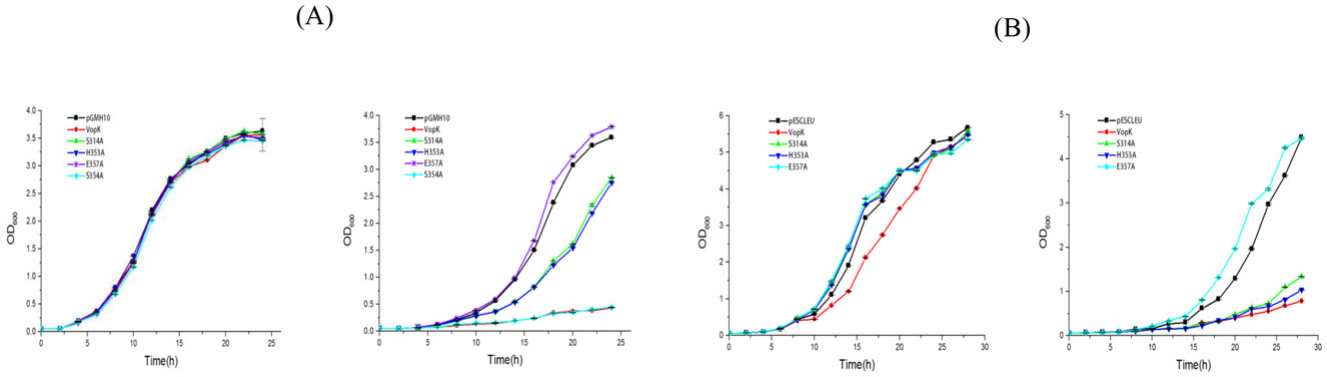
Liquid growth assay. Exponential growth curves demonstrate the effect of wild type and mutant proteins of VopK from pGMH10 (A) or pESC-LEU (B) on BY4741. Cultures were grown in SC media (uninduced, Left panel) and SC^Gal^ media (induced, Right panel). OD_600_ of three technical replicates were tracked. Error bars depict the standard deviation from the mean.

As documented, certain effectors exhibit an altered toxicity in solid and liquid growth assay conditions in yeast model system [18, 20, 30]. To evaluate, we subjected our recombinant yeast strains harboring wild type and alanine derivatives (Ser^314^Ala/His^353^Ala/Glu^357^Ala) of VopK cloned in both low copy (pGMH10) and high copy (pESC-LEU) vectors in liquid growth assay as described elsewhere [7, 15]. In case of recombinant yeast strains harboring wild type and alanine mutants of pGMH10 vector background, we observed an agreement between solid and liquid growth assay results where VopK showed its strong lethality and VopK-E357A variant was non-lethal. On the other hand, VopK-S314A and VopK-H353A maintained partial loss in toxicity (Figs 1B and 4A). In case of recombinant yeast strains carrying wild type and alanine variants of pESC-LEU vector background, we noticed that data are congruent for VopK and VopK-E357A variant in both solid and liquid growth conditions (Figs 1A and 4B). But partial loss in toxicity of VopK-S314A and VopK-H353A are not comparable under these conditions. In liquid growth assay, VopK-S314A and VopK-H353A exhibited little more lethality than that of in solid agar spotting (Figs 1A and 4B). The discrepancy of lethality pattern of VopK-S314A and VopK-H353A variants from pESC-LEU background (high copy number vector) in solid and liquid growth conditions may be explained in terms level of expression under different growth conditions as evidenced earlier [18, 20, 30].

**Fig 5:**
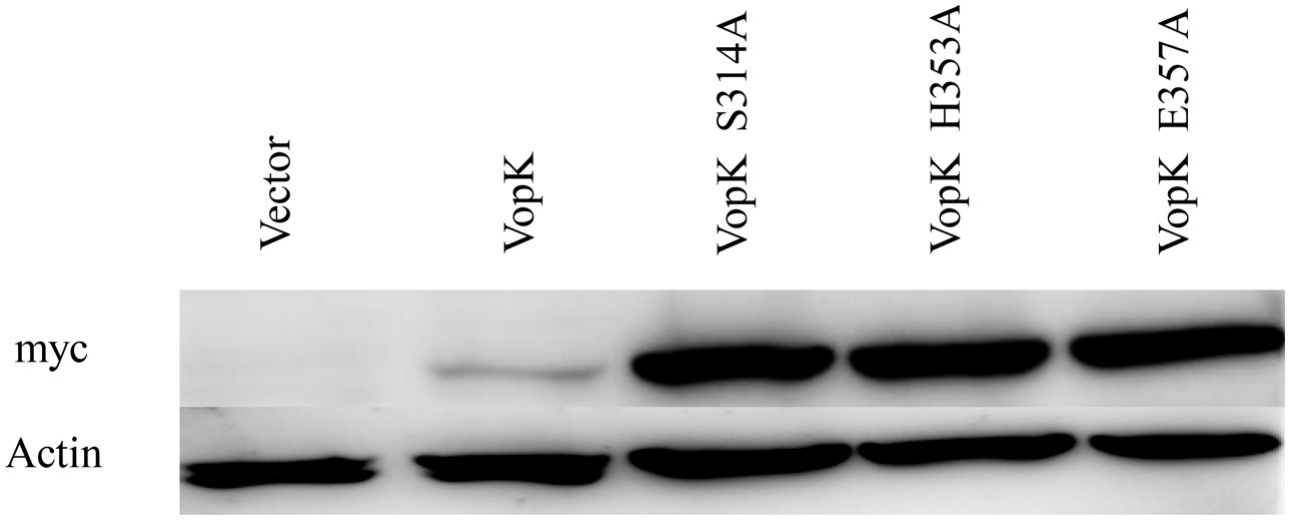
Western blot analysis. Expression of the myc tagged proteins form high copy vector confirmed by immunoblot analysis of whole cell lysates harvested after galactose induction, probed with anti-myc monoclonal antibody. Anti-actin antibodies were used as a loading control (lower panel).

It could be surmised that the loss of lethality (complete and partial) in various VopK alanine congeners (Ser^314^Ala/His^353^Ala/Glu^357^Ala) could be a result of instability of recombinant proteins under *in vivo* condition. To ensure the *in vivo* stability of all alanine congeners of VopK, western blot analysis of recombinant yeast strains harboring wild type and SHE alanine variants (Ser^314^Ala/His^353^Ala/Glu^357^Ala) of VopK in pESC-LEU vector was carried out by using anti-myc antibody as described previously [7]. Our western blot data clearly indicated that all the alanine derivatives such as VopK-S314A, VopK-H353A and VopK-E357A were stable under *in vivo* condition (Fig 5). It should be noted that the stability of wild type protein is relatively less than the corresponding alanine variants. Similar observation has also been recorded earlier where mutants were found more stable than wild type [14, 22, 25].

### Examine the importance of another conserved serine residue at position 354 in the lethality of VopK

Interestingly, there is another conserved serine at position 354 within predicted S^314^H^353^E^357^ region (Fig 2). To ascertain the contribution of serine^354^ residue into the toxicity of VopK, alanine substitution mutagenesis was carried out using pGMH10-VopK as a template and the functionality of corresponding alanine variant was examined in yeast model system. We found that toxicity of VopK-S354A variant remains unaltered as compared to wild type VopK protein, thereby suggesting no contribution of serine at position 354 in the lethality of VopK. Solid agar spotting result was further corroborated by liquid growth assay where similar pattern of growth inhibition was observed (Fig 4A). This data further bolster the importance of serine at position 314 in contributing to the lethality of VopK (Fig 1B).

In essence, present study reveals i) the identification of a predicted MCF1-SHE domain and related catalytic triads in VopK; ii) The lack of in vitro protease activity can be explained in terms of absence of proper in vivo substrates or host factors; iii) Alanine substitution mutagenesis underscores the importance of S^314^H^353^E^357^ in the lethality of VopK in yeast model system where glutamate at position 357 largely contributes in VopK function. The alanine derivative of another conserved serine (Ser^354^) within the predicted SHE domain did not affect the toxicity of VopK.

Multiple lines of converging evidences clearly underpin how evolution contributes to microbial pathogenesis by incorporating unique functional domains and motifs in several bacterial virulence determinants [10, 31]. Integration of unique domains and motifs endows the effector proteins with diverse biochemical activities including (de-) phosphorylation, (de-) ubiquitinylation, adenylate cyclase, AMPylation, ribosylation, lyase reactivity, O-GlcNAcylation and proteolysis [32]. There is now a growing understanding on the evolution of several effector proteins (YopJ, YopT, VopA, AvrA, SseL etc) and polymorphic toxins (such as MCF1, FitD and HopT1-1) as proteases where catalytic triad residues have been evidenced to play a key role in the function of such effectors and large toxins [9–11]. As mentioned in the preceding section, several polymorphic toxins share a novel serine peptidase domain named as MCF1-SHE domain having a catalytic core that comprises serine, histidine and glutamate residues [9]. It is noteworthy to mention that each residue in the catalytic core functions differently in various proteases. For example, chymotrypsin, trypsin and subtilisin contain Ser/His/Asp triad where the contribution of serine and histidine residues is different from that of the aspartic acid toward catalysis [33, 34]. In case of aspartyl dipeptidase protease of *Salmonella typhimurium*, a variation of conventional Ser/His/Asp triad is noticed where aspartate residue is replaced with glutamate [35]. Interestingly, mutagenesis of glutamate residue results in a 100-fold drop in activity whereas mutagenesis of aspartate residue in case of the conventional triad (Ser/His/Asp) results in 10,000-fold reduction in activity [33, 34]. In case of VopK, we observed a complete loss of toxicity in VopK-E357A as compared to other alanine derivatives (e.g VopK-S314A and VopK-H353A), thus indicating differential contribution of triad residues in the functionality of VopK as evaluated by employing yeast model system. In addition, the yeast model of VopK will definitely help to identify cellular pathway (s) targeted by this effector protein which can further be evaluated in a proper mammalian host system. Such model is also useful to screen small molecule inhibitors against this effector molecule. Additional studies are necessary to address these issues.
